# pH and H_2_O_2_ dual-sensitive nanoparticles enable enhanced and safe glucose-responsive oral insulin delivery for diabetes mellitus treatment

**DOI:** 10.7150/thno.98177

**Published:** 2024-09-03

**Authors:** Muzi Li, Nan Wang, Ruiyuan Liu, Xinyue Zhang, Wei He, Wen Zhang, Jiaxin Li, Chen Peng, Yan Li

**Affiliations:** 1 Beijing Key Laboratory for Bioengineering and Sensing Technology, School of Chemistry and Biological Engineering, University of Science and Technology Beijing, Beijing 100083, China.; 2 College of Pharmacy, Heze University, Heze 274015, China.; 3 School of Materials Science and Engineering, University of Science and Technology Beijing, Beijing 100083, China.

**Keywords:** diabetes mellitus, insulin, dual-sensitive glucose-responsive nanoparticles, poly(carboxybetaine), oral administration

## Abstract

**Background:** Oral insulin delivery is considered a revolutionary alternative to daily subcutaneous injection. However, the oral bioavailability of insulin is very low due to the poor oral absorption into blood circulation.

**Methods:** To promote penetration across the intestinal epithelium and achieve enhanced and safe glucose-responsive oral insulin delivery, pH and H_2_O_2_ dual-sensitive nanoparticles (NPs) were constructed. The NPs were loaded of glucose oxidase (GOx) and insulin by pH and H_2_O_2_ dual-sensitive amphiphilic polymer incorporated with phenylboronic ester-conjugated poly(2-hydroxyethyl methacrylate) and poly(carboxybetaine) (PCB). The dual-sensitive NPs were utilized for the treatment of type 1 diabetes mellitus (T1DM) after oral administration.

**Results:** The dual-sensitive NPs could enhance the transport of insulin across the intestinal epithelium into blood facilitated by zwitterionic PCB. By virtue of the generated low pH and high H_2_O_2_ with GOx in hyperglycemic environment, the pH and H_2_O_2_ dual-sensitive NPs were disassembled to achieve rapid and sustained release of insulin. After oral administration of the dual-sensitive NPs in enteric capsules into T1DM mouse model, the oral bioavailability of insulin reached 20.24%, and the NPs achieved hypoglycemic effect for a few hours longer than subcutaneously injected insulin. Importantly, the pH and H_2_O_2_ dual-sensitive NPs could ameliorate the local decline of pH and rise of H_2_O_2_ to avoid the toxic side effect.

**Conclusion:** Therefore, this work would provide a promising platform for the enhanced and safe treatment of diabetes mellitus.

## Introduction

Diabetes mellitus is a metabolic disease associated with elevated blood glucose, which causes a series of complications and seriously threatens the life of patients 1-3. Insulin can control glucose homeostasis by stimulating glucose uptake, and is used for the treatment of type 1 diabetes mellitus (T1DM) and advanced type 2 diabetes mellitus (T2DM) in clinical 4-6. However, insulin is currently limited to the subcutaneous injection. This invasive administration route has the disadvantages of low patient compliance and safety issues 7,8. In comparison, oral administration is a promising alternative route due to its low learning cost, high convenient and patient compliance 9-12. Moreover, oral administration of insulin can provide better glucose homeostasis by mimicking the dynamics of the endogenous insulin 13. However, there are a series of physiological barriers in the gastrointestinal (GI) tract after oral administration, mainly including ultra-acid pH in the stomach, enzymatic degradation, intestinal mucus layer and intestinal epithelial cell layer 14-16. These formidable physiological barriers result in low oral bioavailability and poor therapeutic efficiency of insulin 17-20.

Oral nanoparticles (NPs) have been developed to overcome the barriers in GI tract. In addition to the requirements of the GI environment, other profiles such as glucose-responsiveness should also be considered to mimic the pancreatic β-cell function by releasing insulin according to blood glucose level. Glucose-responsive NPs are expected to overcome the physiological barriers in GI tract and achieve rapid and sustained release of insulin in high blood glucose level 21,22. Glucose oxidase (GOx) is one of the common glucose-responsive elements. GOx can catalyze the oxidation of glucose to gluconic acid in the presence of oxygen, with the generation of H_2_O_2_ 23,24. Glucose-responsive NPs based on GOx always utilized single pH or H_2_O_2_-sensitive element to promote the release of insulin. However, a local decline of pH or rise of H_2_O_2_ would cause safety problems.

**Scheme 1:**
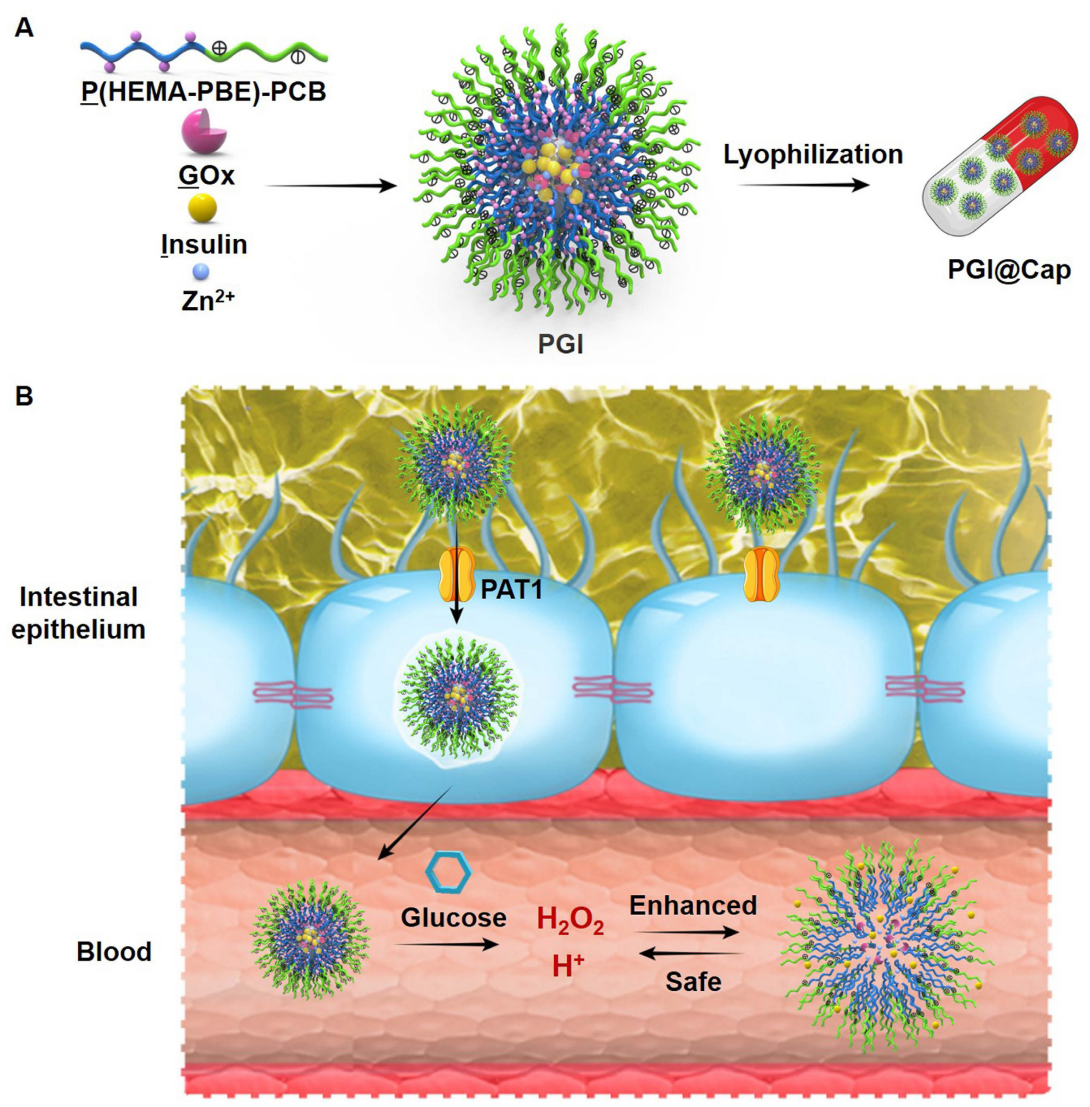
** Schematic illustration of the pH and H_2_O_2_ dual-sensitive NPs. (A)** Preparation of the dual-sensitive NPs. **(B)** Mechanism of the dual-sensitive NPs for enhanced and safe glucose-responsive oral insulin delivery for diabetes mellitus treatment.

Herein, we developed pH and H_2_O_2_ dual-sensitive NPs for enhanced and safe glucose-responsive oral insulin delivery. As shown in Scheme 1A, a pH and H_2_O_2_ dual-sensitive amphiphilic polymer incorporated with phenylboronic ester (PBE)-conjugated poly(2-hydroxyethyl methacrylate) (PHEMA) and poly(carboxybetaine) (PCB) (designated P(HEMA-PBE)-PCB) was synthesized for the encapsulation of GOx and insulin. The hydrophobic block P(HEMA-PBE) could lose the PBE side chain induced by H_2_O_2_ and become hydrophilic, leading to the disassemble of the NPs 25. The hydrophilic block zwitterionic PCB is electrically neutral composed of cationic quaternary amine groups and anionic carboxylate groups 26. On the one hand, zwitterionic PCB could enhance the transport of insulin across the intestinal epithelium due to its binding ability with proton-assisted amino acid transporter 1 (PAT1) on epithelial cell layer 27. On the other hand, the negatively charged carboxylate groups of PCB could be protonated at acidic environment 28-30. The electrostatic repulsion of protonated PCB further promoted the release of insulin in high blood glucose level. More importantly, the pH and H_2_O_2_ dual-sensitive amphiphilic polymer P(HEMA-PBE)-PCB could ameliorate the local decline of pH and rise of H_2_O_2_ to achieve safe treatment of diabetes mellitus.

The dual-sensitive NPs encapsulating GOx and insulin were named PGI. To enhance the stability of NPs in stomach, the lyophilized NPs were placed into enteric capsules to obtain PGI@Cap oral dosage. Enteric capsules remain intact to protect the NPs from harsh environment in stomach and rapidly dissolve to release the NPs in small intestine 31. The mechanism of PGI@Cap for diabetes mellitus treatment was shown in Scheme 1B. After oral administration, the PGI NPs reached the small intestine with the protection of enteric capsules. The PGI NPs crossed the intestinal epithelium into blood via the recognition between PAT1 and PCB. By virtue of the generated low pH and high H_2_O_2_ with GOx in hyperglycemic environment, the dual-sensitive PGI NPs were disassembled to achieve rapid and sustained release of insulin for enhanced diabetes mellitus treatment. In the meantime, the dual-sensitive NPs consumed the H^+^ and H_2_O_2_, which could ameliorate the local decline of pH and rise of H_2_O_2_ for safe diabetes mellitus treatment.

## Methods

### Materials

4-Dimethylaminopyridine (DMAP), 2-hydroxyethyl methacrylate (HEMA), GOx and betaine were purchased from J&K scientific (Beijing, China). 2-Cyano-2-propyl benzodithioate (CPB) and 2,2'-azobis(2-methylpropionitrile) (AIBN) were from Aladdin (Shanghai, China). N,N'-Carbonyldiimidazole (CDI) and 4-(hydroxymethyl)phenylboronic acid pinacol ester (PAPE) were obtained from Energy Chemical (Shanghai, China). 1-(3-Dimethylaminopropyl)-3-ethylcarbodiimide hydrochloride (EDC·HCl) was from Boer Chemical Regents (Shanghai, China). 4',6-Diamidino-2-phenylindole (DAPI), 4% paraformaldehyde, GOx activity assay kit, dulbecco's modified eagle medium (DMEM) (without glucose) and goat anti-rabbit lgG(H+L)-Fluor594 conjugated secondary antibody were purchased from Solarbio (Beijing, China). Fetal bovine serum (FBS) was from Cyagen (Jiangsu, China). 3-(4,5-Dimethylthiahiazol-2-yl)-2,5-diphenytetrazolium bromide (MTT) was from Lablead (Beijing, China). Enteric capsule was purchased from Yuyan Instruments Co., Ltd (Shanghai, China). Anti-claudin-4 primary antibody was obtained from Affinity Biosciences LTD. Mouse insulin ELISA kit was purchased from Abcam (Ab277390). Mouse TNF-α ELISA kit and mouse IL-6 ELISA kit were from MultiSciences (LiankeBio).

### Synthesis of P(HEMA-PBE)-PCB

Carboxybetaine (CB) and PCB with the theoretical degree of polymerization (DP) of 50 were synthesized according to our previous study 15. PHEMA-PCB was synthesized by the reversible addition-fragmentation chain transfer polymerization (RAFT). As the theoretical DP of 50 for PHEMA as an example, PCB (105.3 mg 0.02 mmol), HEMA (130.1 mg, 1 mmol) and AIBN (26.3 mg) were dissolved in DMSO. The system was degrassed by three freeze-pump-thaw cycles and backfilled with nitrogen. After 24 h of reaction at 60 °C, the solution was dialyzed in dialysis bag (MWCO 3,500) against deionized water for 48 h to remove the unreacted monomers. The final product was obtained by freeze-drying. Polymers with the theoretical DP of 25 and 100 for PHEMA were synthesized by the same method. ^1^H NMR was carried out to characterize the obtained products.

4-(Imidazoyl carbamate)phenylboronic acid pinacol ester was synthesized with PAPE and CDI according to the literature 25. ^1^H NMR (Bruker 400 MHz, CDCl_3_, δ ppm) was carried out to characterize the obtained product. PHEMA-PCB (40.0 mg, OH 0.14 mmol), 4-(imidazoyl carbamate)phenylboronic acid pinacol ester (76.9 mg, 0.21 mmol), and DMAP (25.6 mg, 0.21 mmol) were dissolved in 5 mL of anhydrous DMSO. After 12 h of reaction, the solution was dialyzed in dialysis bag (MWCO 3,500) against ethanol and deionized water for 12 h to remove the unreacted monomers, respectively. The final product was obtained by freeze-drying. ^1^H NMR (Bruker 400 MHz, DMSO-d6, δ ppm) was carried out to characterize the obtained products.

### Preparation and characterization of PGI NPs

P(HEMA-PBE)-PCB was dissolved in DMSO at a concentration of 20 mg/mL. P(HEMA-PBE)-PCB was then added into a solution containing 0.5 mg of insulin and 0.125 mg of GOx. 150 μL of ZnCl_2_ (1 mg/mL) solution was added dropwise to the above solution and stirred for 5 min. The resulting mixture was dialyzed in dialysis bag (MWCO 7,000) against deionized water for 2 h to obtain the PGI NPs. PI NPs were prepared by the same method without GOx. The particle size and zeta potential of the NPs were determined using dynamic light scattering (DLS) (Zetasizer Nano ZS90, UK). NPs were added dropwise to carbon-coated 230-mesh copper grids. After drying with infrared light, the morphology of NPs was observed by transmission electron microscope (TEM, JEM 1200EX, Japan). The drug loading efficiency was calculated by destroying the NPs loading of Cy5-insulin with 10% methanol. The GOx loading efficiency was calculated by the activity assay kit. The fluorescence intensity was detected with a microplate reader (Molecular Devices, SpectraMax iD3).



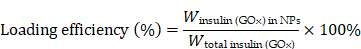



### Lyophilized stability of PGI NPs

The particle solution was lyophilized, and then the diameter and zeta potential were measured by DLS after redissolving. The morphology of NPs after lyophilization was observed by TEM.

### Motion behavior in mucus

Mucus was reconstituted by dissolving porcine stomach mucin in PBS at 20 mg/mL. Cy5-insulin, P_17_GI-10 and P_17_GI-15 NPs with Cy5-insulin were added into 1 mL of mucus to reach a final concentration of 1% v/v, respectively. Videos were captured with a ×10 oil-immersion objective in high-speed camera (PCO DIMAX HS1 High Speed CMOS Camera). For each sample, trajectories of thirty particles were analyzed, and three samples were examined. The mean square displacement (MSD) was calculated with the equation of MSD = <r(t)^2^-r(0)^2^> at a temporal resolution of 500 ms for 2 s.

### Dual-sensitive ability of PGI NPs

PGI NPs were mixed with 100 mg/dL and 400 mg/dL of glucose solution, respectively. The concentration of GOx was 6.25 μg/mL. The pH value of the solution was then measured with a pH Meter (INESA PHSJ-4F, China) at different time points. The concentration of H_2_O_2_ in the solution was detected with the ELISA kit. 30% H_2_O_2_ and P(HEMA_17_-PBE)-PCB_22_ (20 mg/mL) were mixed 4: 1 (v/v) and reacted for 24 h. The solution was dialyzed in dialysis bag (MWCO 3,500) against deionized water for 12 h and freeze-dried to obtain the product. ^1^H NMR (Bruker 400 MHz, DMSO-d6, δ ppm) was carried out to characterize the obtained product.

### *In vitro* drug release

PGI NPs with Cy5-insulin in dialysis bag (MWCO 7,000) were incubated in 30 mL of intestinal simulated fluid, 100 mg/dL and 400 mg/dL of glucose solution at 37 °C under agitation, respectively. At the set time points, 400 μL solution was removed and the same volume of fresh solution was added. The fluorescence intensity was detected by microplate reader. The release profile was calculated by formula:



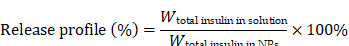



### Cytotoxicity

Caco-2 cells were incubated with DMEM (without glucose) containing 20% FBS, 1% penicillin (100 units/mL) and streptomycin (100 μg/mL) at 37 °C in a 5% CO_2_ atmosphere. To determine the cytotoxicity of the NPs, different concentrations of free insulin, P(HEMA_17_-PBE)-PCB_22_ and P_17_GI-15 NPs were added to each 96-well plates, respectively. HEK293 cells were incubated with DMEM (high glucose), 10% FBS and 1% penicillin (100 units/mL) and streptomycin (100 μg/mL). Cells seeded in 96-well plates were treated with free GOx and P_17_GI-15 NPs with GOx concentration of 0.33 μg/mL, respectively. After 24 h of incubation, the cytotoxicity was detected via MTT assay. The cell viability of untreated cells was defined as 100%.

### Cellular uptake

To detect the cellular uptake and probe the mechanism of the dual-sensitive P_17_GI-15 NPs across the intestinal epithelium, the cellular uptake study was performed at 4 °C or in the presence of various inhibitors. Caco-2 cells seeded in 12-well plates were preincubated with betaine (2 mg/mL) or endocytic inhibitors including methyl-β-cyclodextrin (mβCD, 50 μM), chlorpromazine (10 μg/mL) and wortmannin (20 μg/mL) for 1 h before the addition of P_17_GI-15 NPs with Cy5-insulin (insulin 1.33 μg/mL), respectively. After 4 h of incubation, cells were harvested in PBS and assessed with BD Caliburflow cytometry (BD CO., USA) to determine the mean fluorescence intensity of Cy5.

### Immunofluorescence staining and Western blot analysis

Caco-2 cells were cultured in Petri dishes and treated with PBS and P_17_GI-15 NPs (insulin 1.33 μg/mL) for 4 h, respectively. Cells were stained with anti-claudin-4 primary antibody and DAPI in sequence according to our previous study 15. After three times washing with PBS, cells were then observed with confocal laser scanning microscope (CLSM). The level of claudin-4 (CLDN4) was also measured by Western blot using the chemiluminescence analyzer (Tanon MINI SPACE 2000, China). The ImageJ software was used for analysis.

### Transport of P_17_GI-15 NPs across Caco-2 cell monolayer

Caco-2 cells were cultured in 24-well transwell plates and used when resistance across the insert membrane reached at least 300 Ω cm^2^. The basal chamber medium was replaced with DMEM (without glucose) containing 20% FBS, and the apical medium was replaced with blank, Cy5-insulin, P_17_GI-15 NPs with Cy5-insulin, respectively. At designed time points, 100 μL of culture medium in the basement chamber was taken, and 100 μL of fresh culture medium was supplemented at the same time. The fluorescence intensity of Cy5 was detected by microplate reader. The apparent permeability coefficient (*P*_app_) was calculated by the following equation.



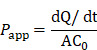



The dQ/dt was the amount of insulin transport to basolateral side per second. A meant the diffusion area of the monolayer (0.33 cm^2^), and C_0_ was the initial insulin concentration.

### Biodistribution of P_17_GI-15 NPs *in vivo* after oral administration

All animal experiments in this study were performed in accordance with protocols approved by the Institutional Animal Care and Use Committee (IACUC-AMSS-20240103-01). ICR mice were fasted for 6 h before administration. Saline, Cy5-insulin, P_17_GI-15 NPs and P_17_GI-15@Cap were orally administered to mice, respectively. After 4 h of administration, mice were euthanized, and tissues were harvested. The biodistribution of mice was detected via a PerkinElmer IVIS Spectrum *in vivo* imaging system.

### Pharmacokinetic and hypoglycemic effect* in vivo*

For the T1DM induction, ICR mice were injected with streptozotocin (40 mg/kg) once a day for five days via the intraperitoneal injection. The fasting blood glucose level of mice reached at least 16.0 mM were considered to be diabetic. Saline, free insulin solution, P_17_GI-15@Cap and P_17_I-15@Cap at an insulin dose of 50 IU/kg were given by gavage, respectively. One group of diabetic mice were subcutaneous injection with insulin solution at a dose of 5 IU/kg. At designed time points, blood glucose levels were quantified with a blood glucose meter. Plasma insulin levels were measured using the ELISA kit. The oral bioavailability (*F*%) of insulin was calculated using the following equation. The area under the curve (AUC) was calculated from the plasma insulin concentration curve. After 24 h of administration, blood samples were collected to detect the pro-inflammatory cytokines. Mice were euthanized and small intestines were harvested for hematoxylin-eosin staining.



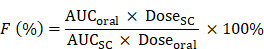



An intraperitoneal glucose tolerance test (IPGTT) was further performed to evaluate the effectiveness of the P_17_GI-15@Cap. After 4 h of treatment, glucose solution (1.5 g/kg) was intraperitoneal injected, and the changes of blood glucose level were quantified with a blood glucose meter.

To verify the long-term therapeutic efficacy, saline and P_17_GI-15@Cap at an insulin dose of 50 IU/kg were orally administered to STZ-induced diabetic mice once a day for five consecutive days, respectively. One group of diabetic mice were subcutaneous injection with insulin solution at a dose of 5 IU/kg. After 2 h of administration, mice were re-feeding for 16 h. At designed time points, blood glucose level was determined using a blood glucose meter. After 24 h of the last administration, blood samples were collected to detect the pro-inflammatory cytokines, and the small intestines were also harvested for hematoxylin-eosin staining.

### Statistical analysis

All data were presented as mean ± SD. Statistical significance was analyzed using a two-tailed *t*-test between 2 groups. An ANOVA with Tukey's test was carried out to analyze the statistical significance between more than 2 groups. Statistical analysis was carried out using graphpad prism 8.0.1 software (*P < 0.05, **P < 0.01, ***P < 0.001).

## Results and Discussion

### Preparation and characterization of the dual-sensitive NPs

PCB and PHEMA-PCB were synthesized via the RAFT (Figure S1). ^1^H NMR spectra indicated the successful synthesis of CB monomer (Figure S2) and PCB (Figure S3). The DP of PCB was determined by gel permeation chromatography (GPC). PCB with the weight-averaged molecular weight of 5263 was obtained (Figure S4), and its corresponding DP was approximately 22. The chemical structure of PHEMA-PCB was characterized by ^1^H NMR (Figure S5), which indicated the successful synthesis of PHEMA_17_-PCB_22_, PHEMA_34_-PCB_22_ and PHEMA_44_-PCB_22_ determined by using the peak area with the PCB as a standard, respectively. Furthermore, 4-(imidazoyl carbamate)phenylboronic acid pinacol ester was successfully synthesized (Figure S6), and PBE was conjugated to the hydroxyl groups of PHEMA via a carbonate linkage. Character peaks of PBE in ^1^H NMR spectra indicated the successful conjugation (Figure S7).

**Figure 1:**
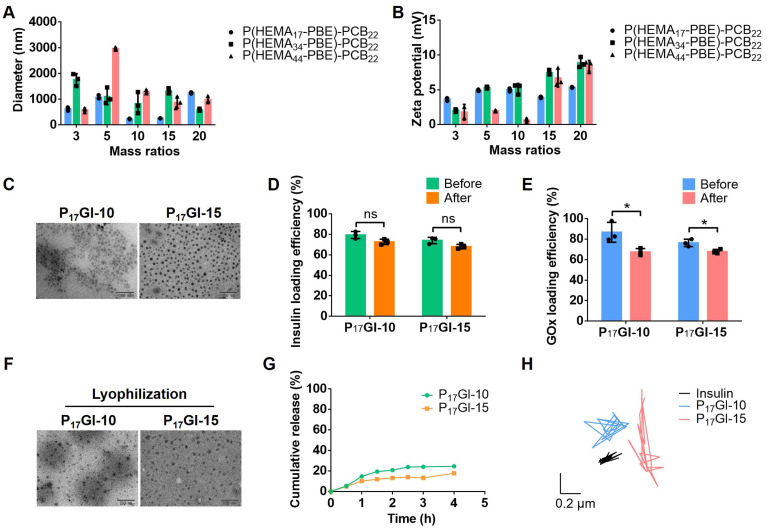
** Characterization of the dual-sensitive NPs. (A)** Hydrodynamic diameter and **(B)** zeta potential of PGI NPs with different mass ratios between P(HEMA-PBE)-PCB and insulin. **(C)** TEM images of P_17_GI-10 and P_17_GI-15 NPs. Scale bar: 200 nm. **(D)** Insulin and **(E)** GOx loading efficiency of NPs before and after lyophilization. **(F)** TEM images of P_17_GI-10 and P_17_GI-15 NPs after lyophilization. Scale bar: 200 nm. **(G)** Cumulative insulin release of P_17_GI-10 and P_17_GI-15 NPs in intestinal simulated fluid. **(H)** Representative motion trajectories of the P_17_GI-10 and P_17_GI-15 NPs with Cy5-insulin in mucus at a time lapse of 2 s. Data are presented as the mean ± SD (n = 3). ns > 0.05, *P < 0.05.

The PGI NPs were prepared via Zn^2+^ precipitation method. Firstly, ZnCl_2_ solution was slowly added to the mixture of insulin and GOx under stirring. The smallest hydrodynamic diameter was about 238.5 nm at mass ratio of 0.3 between ZnCl_2_ and insulin (Figure S8A). The corresponding zeta potential was -18.2 mV (Figure S8B). Next, at this mass ratio, PGI NPs were prepared via the slow addition of ZnCl_2_ solution to the mixture of insulin and GOx in the presence of P(HEMA-PBE)-PCB. At mass ratio of 10 and 15 between P(HEMA_17_-PBE)-PCB_22_ and insulin, the hydrodynamic diameters of the obtained PGI NPs were around 200 nm (Figure 1A) with polydispersity index (PDI) of 0.30 and 0.34 (Figure S9), respectively. The zeta potential of these two NPs was close to neutral (Figure 1B). These two PGI NPs named P_17_GI-10 and P_17_GI-15 NPs possessed the desired particle size and zeta potential for diffusion through the mucus layer 32,33, thereby choosing for the following studies. The morphology of NPs was characterized by TEM that P_17_GI-10 and P_17_GI-15 NPs with spherical structure were obtained (Figure 1C). The size of both NPs observed in Figure 1C was smaller than that measured by DLS, which might be due to the dehydration during sample preparation in TEM.

The corresponding non-glucose-responsive NPs without GO_X_ were prepared via the same method. The hydrodynamic diameter of P_17_I-10 NPs was 168.2 nm with the zeta potential of -0.72 mV, and it was 254.0 nm for P_17_I-15 NPs with the zeta potential of -2.99 mV (Figure S10). The insulin loading efficiencies were above 68% for all four NPs (Figure 1D and Figure S11). The GOx loading efficiencies were determined as 86.7% and 76.3% for P_17_GI-10 and P_17_GI-15 NPs, respectively (Figure 1E).

To prevent the degradation of insulin in the acidic gastric juice, PGI NPs were lyophilized, and the powder was expected to be loaded into enteric capsules. The hydrodynamic diameter of P_17_GI-10 and P_17_GI-15 NPs showed negligible changes after lyophilization (Figure S12). As shown in Figure 1D, the loading efficiencies of insulin had no significant reduction after lyophilization. The GOx activities and corresponding loading efficiencies were reduced after lyophilization (Figure 1E and Figure S13). The NPs maintained their original morphology after lyophilization observed via TEM (Figure 1F), which was consistent with the results from hydrodynamic diameter.

Furthermore, the cumulative insulin release of P_17_GI-10 and P_17_GI-15 NPs has been detected in intestinal simulated fluid, respectively. As shown in Figure 1G, the cumulative release percentages of insulin were 24.5% and 17.7% for P_17_GI-10 NPs and P_17_GI-15 NPs after 4 h incubation, respectively. These results indicated their good stability in intestinal simulated fluid.

Mucus covers the intestinal epithelium, and can rapidly trap the foreign particles 34. The movement behavior of P_17_GI-10 and P_17_GI-15 NPs in mucus was recorded with a ×10 oil-immersion objective in high-speed camera, and the MSD was calculated. As shown in Figure 1H and Figure S14, both NPs spanned much longer distance than free Cy5-insulin at a time scale of 2 s. This was beneficial for the oral delivery of insulin across the mucus (Figure S15).

### Glucose-responsive behavior of the dual-sensitive NPs

The glucose-responsive ability of PGI NPs was assessed *in vitro* in response to different glucose levels. GOx, a glucose-specific enzyme, catalyzes glucose and oxygen to gluconic acid and H_2_O_2_ (Figure S16A) 35. ^1^H NMR spectrum confirmed that P(HEMA_17_-PBE)-PCB_22_ polymer lost the PBE side chain induced by H_2_O_2_ (Figure S16B).

**Figure 2:**
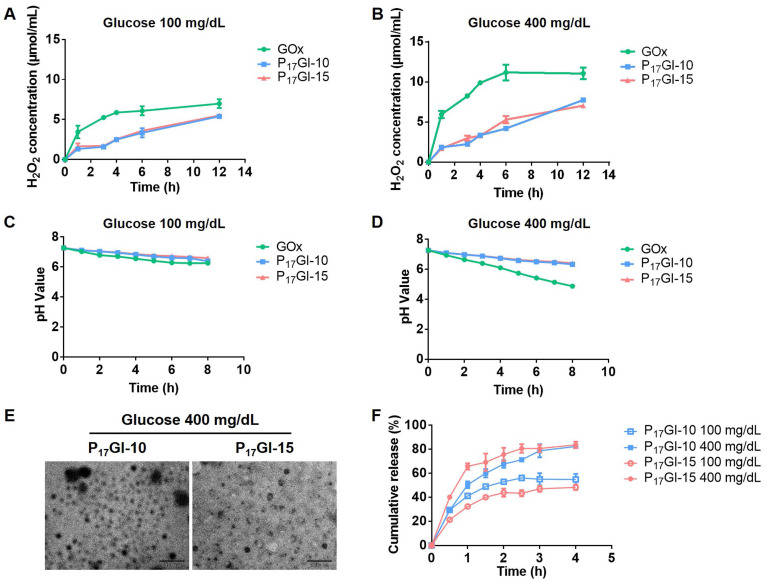
** Glucose-responsive behavior of the dual-sensitive NPs.** The concentrations of H_2_O_2_ in **(A)** 100 mg/dL and **(B)** 400 mg/dL glucose solution after free GOx and PGI NPs incubation. The pH values in **(C)** 100 mg/dL and **(D)** 400 mg/dL glucose solution after free GOx and PGI NPs incubation. **(E)** TEM images of P_17_GI-10 and P_17_GI-15 NPs in 400 mg/dL glucose solution. Scale bar: 200 nm. **(F)** Cumulative insulin release of P_17_GI-10 and P_17_GI-15 NPs in 100 mg/dL and 400 mg/dL glucose solution, respectively. Data are presented as the mean ± SD (n = 3).

Furthermore, the changes of pH and H_2_O_2_ level in different glucose solutions were detected after treatment with GOx and PGI NPs, respectively. The concentrations of glucose were 100 mg/dL and 400 mg/dL to simulate a normoglycemic level and typical hyperglycemic level, respectively. As shown in Figure 2A and 2B, GOx increased the level of H_2_O_2_ in 100 mg/dL and 400 mg/dL glucose solutions, while P_17_GI-10 and P_17_GI-15 NPs could obviously reduce the concentration of H_2_O_2_. In addition, the pH decreased to be as low as 4.88 at the hyperglycemic level (Figure 2C and 2D). In comparison, P_17_GI-10 and P_17_GI-15 NPs could ameliorate the decline of pH value, and the pH value was 6.42 for P_17_GI-15 NPs at the hyperglycemic level after 8 h incubation. These results verified that the pH and H_2_O_2_ dual-sensitive amphiphilic polymer P(HEMA-PBE)-PCB in NPs could ameliorate the local decline of pH and rise of H_2_O_2_ generated via GOx in glucose solution.

The changes of hydrodynamic diameter and zeta potential of PGI NPs were measured after incubation in 400 mg/dL glucose solution (Figure S17). The diameter of both NPs obviously increased to approximately 500 nm, and the zeta potential also significantly increased to 12.5 mV and 23.8 mV for P_17_GI-10 and P_17_GI-15 NPs, respectively. The TEM images confirmed the dissociation of P_17_GI-10 and P_17_GI-15 NPs after incubation in 400 mg/dL glucose solution, and NPs became large and small particles (Figure 2E).

The cumulative insulin release of PGI NPs and PI NPs was further evaluated at different glucose levels. The cumulative release percentages of insulin had no obvious difference for P_17_I-10 and P_17_I-15 NPs in 100 mg/dL and 400 mg/dL glucose solution, respectively (Figure S18). The cumulative release percentages of insulin were approximately 24.0% for P_17_I-15 NPs at the normoglycemic and hyperglycemic level after 1 h incubation. In contrast, a rapidly insulin release was observed at the hyperglycemic level for PGI NPs (Figure 2F). The cumulative release percentages of insulin were 50.5% and 65.8% for P_17_GI-10 and P_17_GI-15 NPs at the hyperglycemic level after 1 h incubation, respectively, which were higher than that of both PGI NPs at the normoglycemic level at the same time point. These results demonstrated that the glucose-responsive PGI NPs could achieve rapidly insulin release at the hyperglycemic level. In addition, at the same time points, the cumulative release percentages of P_17_GI-15 NPs at the normoglycemic level were lower than that of P_17_GI-10 NPs at the normoglycemic level. The slowly insulin release behavior of P_17_GI-15 NPs at the normoglycemic level was benefit for safety therapy. Therefore, the dual sensitive P_17_GI-15 NPs were chosen for the following studies due to their better insulin release behavior.

### Biocompatibility and penetration ability of the dual-sensitive NPs *in vitro*

**Figure 3:**
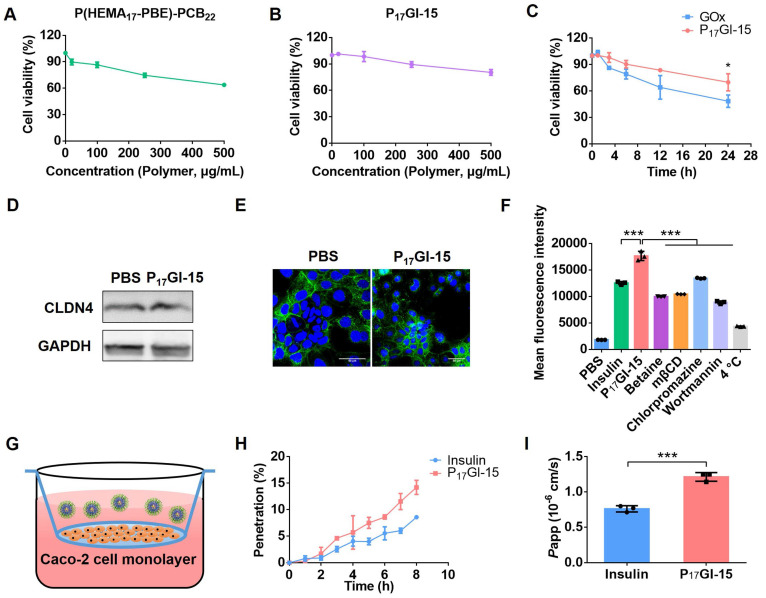
** Biocompatibility and penetration ability of the dual-sensitive NPs* in vitro*.** Cytotoxicity of** (A)** P(HEMA_17_-PBE)-PCB_22_ polymer and **(B)** P_17_GI-15 NPs on Caco-2 cells cultured with no glucose medium. **(C)** Cytotoxicity of GOx and P_17_GI-15 NPs on HEK293 cells cultured with high glucose medium. CLDN4 levels detected by **(D)** Western blotting and **(E)** CLSM. Scale bar: 50 μm. **(F)** The cellular uptake of free Cy5-insulin and P_17_GI-15 NPs detected by flow cytometry. Mechanistic probe was performed by monitoring the cellular uptake level at 4 °C or in the presence of various inhibitors.** (G)** The transwell model. **(H)** The percentage of Cy5-insulin across the Caco-2 cell monolayer in transwell. **(I)** The *P*_app_ values of free Cy5-insulin and P_17_GI-15 NPs. Data are presented as the mean ± SD (n = 3). *P < 0.05, **P < 0.01, ***P < 0.001.

Caco-2 cells, an *in vitro* model of small intestine, were utilized for *in vitro* experiments 36. MTT assay showed that free insulin had a growth promoting effect on Caco-2 cells at the testing concentrations (Figure S19). The cell viability was as high as 86.6% for P(HEMA_17_-PBE)-PCB_22_ polymer at the concentration of 100 μg/mL (Figure 3A), indicating its good biocompatibility. To mimic the fasting state before oral administration, Caco-2 cells were cultured with P_17_GI-15 NPs in no glucose medium for 24 h. The cell viability reached 98.4% at 100 μg/mL P(HEMA_17_-PBE)-PCB_22_ polymer (Figure 3B). Furthermore, to evaluate the biocompatibility of P_17_GI-15 NPs at the hyperglycemic level, HEK293 cells were cultured with high glucose medium, and then incubated with free GOx and P_17_GI-15 NPs, respectively. As shown in Figure 3C, GOx induced the cytotoxicity due to its reaction with glucose, while the pH and H_2_O_2_ dual-sensitive P_17_GI-15 NPs could significantly reduce its cytotoxicity via their ability of ameliorating the local decline of pH and rise of H_2_O_2_.

Epithelial cells are bound by tight junctions 37. To detect the tight junctions formed by Caco-2 cells after P_17_GI-15 NPs treatment, the expression level of CLDN4 was examined via Western blot. As shown in Figure 3D and Fiugre S20, the expression level of CLDN4 had no significant change between P_17_GI-15 NPs and PBS treated cells. Similar results were observed by CLSM (Figure 3E). Therefore, the P_17_GI-15 NPs had negligible damage to the tight junctions.

PAT1, highly expressed on epithelial cell, is known for facilitating the penetration of zwitterionic PCB-based NPs across the epithelial cell layer 27. The cellular uptake was detected by flow cytometry. As shown in Figure 3F, compared with free Cy5-insulin, Cy5-insulin for P_17_GI-15 NPs exhibited increased uptake by Caco-2 cells. Furthermore, we probed the mechanism of the dual-sensitive P_17_GI-15 NPs across the intestinal epithelium by detecting the cellular uptake study at 4 °C or in the presence of various inhibitors. Once Caco-2 cells were pretreated with betaine, P_17_GI-15 NPs exhibited significantly reduced endocytosis efficiency. These results demonstrated that P_17_GI-15 NPs could significantly enhance the transepithelial transport efficiency of insulin facilitated by PAT1. mβCD, chlorpromazine and wortmannin significantly inhibited the cellular uptake level, indicating that P_17_GI-15 NPs were internalized via caveolae/clathrin-mediated endocytosis and macropinocytosis pathway 38. In addition, the cellular internalization of P_17_GI-15 NPs was energy-dependent process as the cellular uptake was significantly blocked at 4 °C.

Moreover, Caco-2 cell monolayer in transwell was utilized to evaluate the penetration ability of P_17_GI-15 NPs across the epithelial cell layer (Figure 3G). Compared with free Cy5-insulin, P_17_GI-15 NPs enhanced the percentage of Cy5-insulin across the Caco-2 cell monolayer (Figure 3H). Furthermore, the *P*_app_ value of Cy5-insulin for P_17_GI-15 NPs was significantly higher than that of free Cy5-insulin (Figure 3I). These results indicated that P_17_GI-15 NPs could significantly enhance the transepithelial transport efficiency of insulin facilitated by PAT1. In addition, the structure of P_17_GI-15 NPs across the Caco-2 cell lay in transwell was observed by TEM. As shown in Figure S21, most of the NPs retained the intact structure.

### Biodistribution of the dual-sensitive NPs *in vivo*

**Figure 4:**
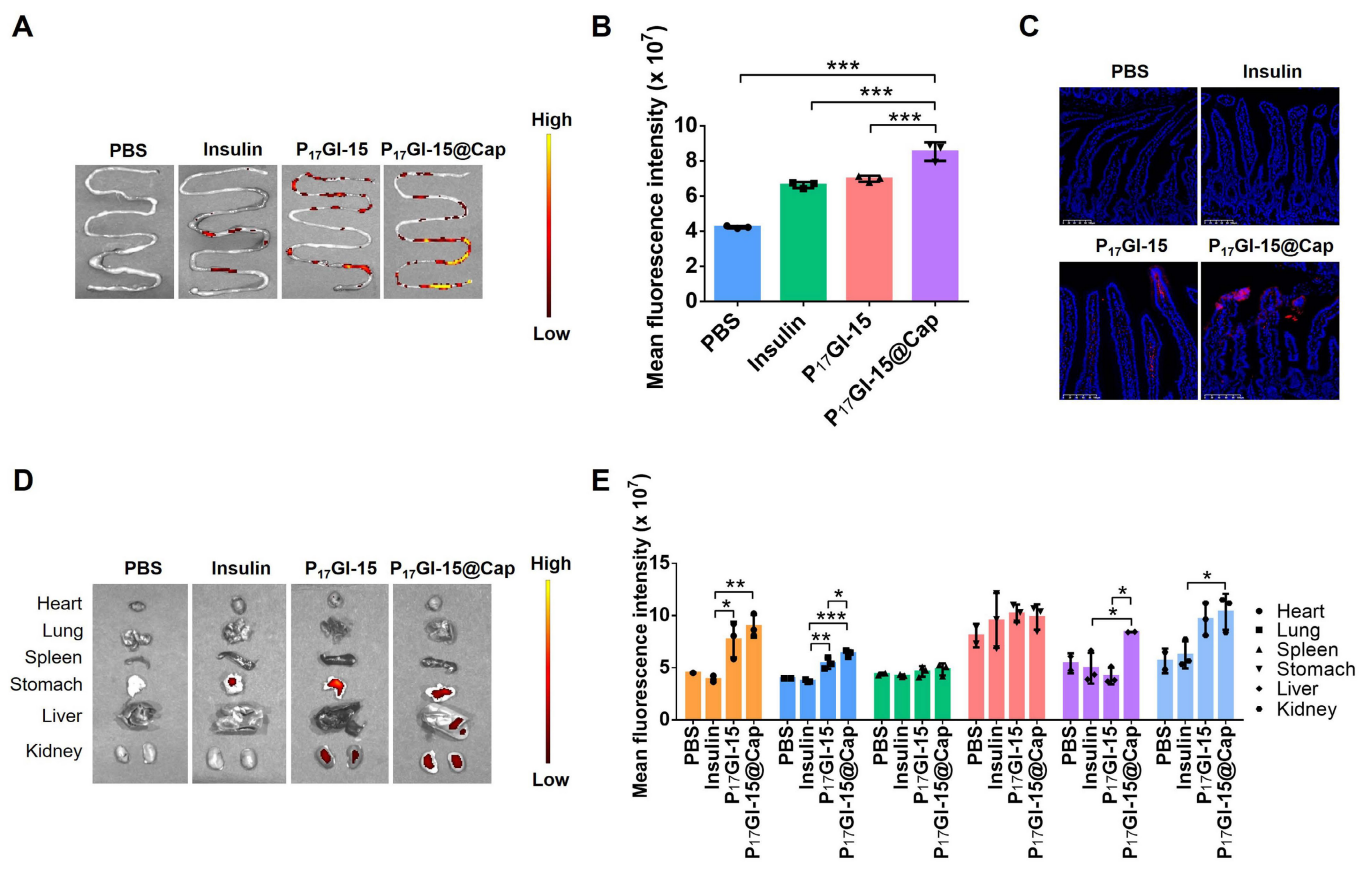
** Biodistribution of the dual-sensitive NPs *in vivo*. (A)** *Ex vivo* biodistribution and **(B)** fluorescence analysis of small intestines after 4 h of oral administration of free Cy5-insulin, P_17_GI-15 NPs and P_17_GI-15@Cap with Cy5-insulin. **(C)** Fluorescence images of small intestine tissue sections after 4 h of oral administration of different formulations. The red fluorescence was from Cy5-insulin. Scale bar: 100 μm. **(D)** *Ex vivo* biodistribution and **(E)** fluorescence analysis of other organs after 4 h of oral administration. Data are presented as the mean ± SD (n = 3). *P < 0.05, **P < 0.01, ***P < 0.001.

We further explored the absorption of the P_17_GI-15 NPs in small intestine after oral administration via an *ex vivo* fluorescence imaging study. Free Cy5-insulin, P_17_GI-15 NPs and P_17_GI-15@Cap were administered through oral gavage. Results showed that mice treated with P_17_GI-15@Cap showed the highest mean fluorescence intensity after 4 h oral administration (Figure 4A and 4B). The small intestine section of the P_17_GI-15@Cap group exhibited strong red fluorescence, which confirmed the high small intestinal absorption of Cy5-insulin (Figure 4C). These results proved that P_17_GI-15@Cap could significantly improve the retention and absorption of Cy5-insulin in the small intestine.

Other organs were also collected and characterized via an *ex vivo* fluorescence imaging study. Organs including heart, liver, lung and kidney from the group of P_17_GI-15@Cap showed the highest fluorescence intensity compared with other groups (Figure 4D and 4E). The enrichment of Cy5-insulin in these organs confirmed the successful transportation of the insulin into the systematic circulation through the GI tract. These results suggested the superiority of P_17_GI-15@Cap as a vehicle for promoting oral insulin delivery.

### Pharmacokinetic and hypoglycemic effect of the dual-sensitive NPs* in vivo*

**Figure 5:**
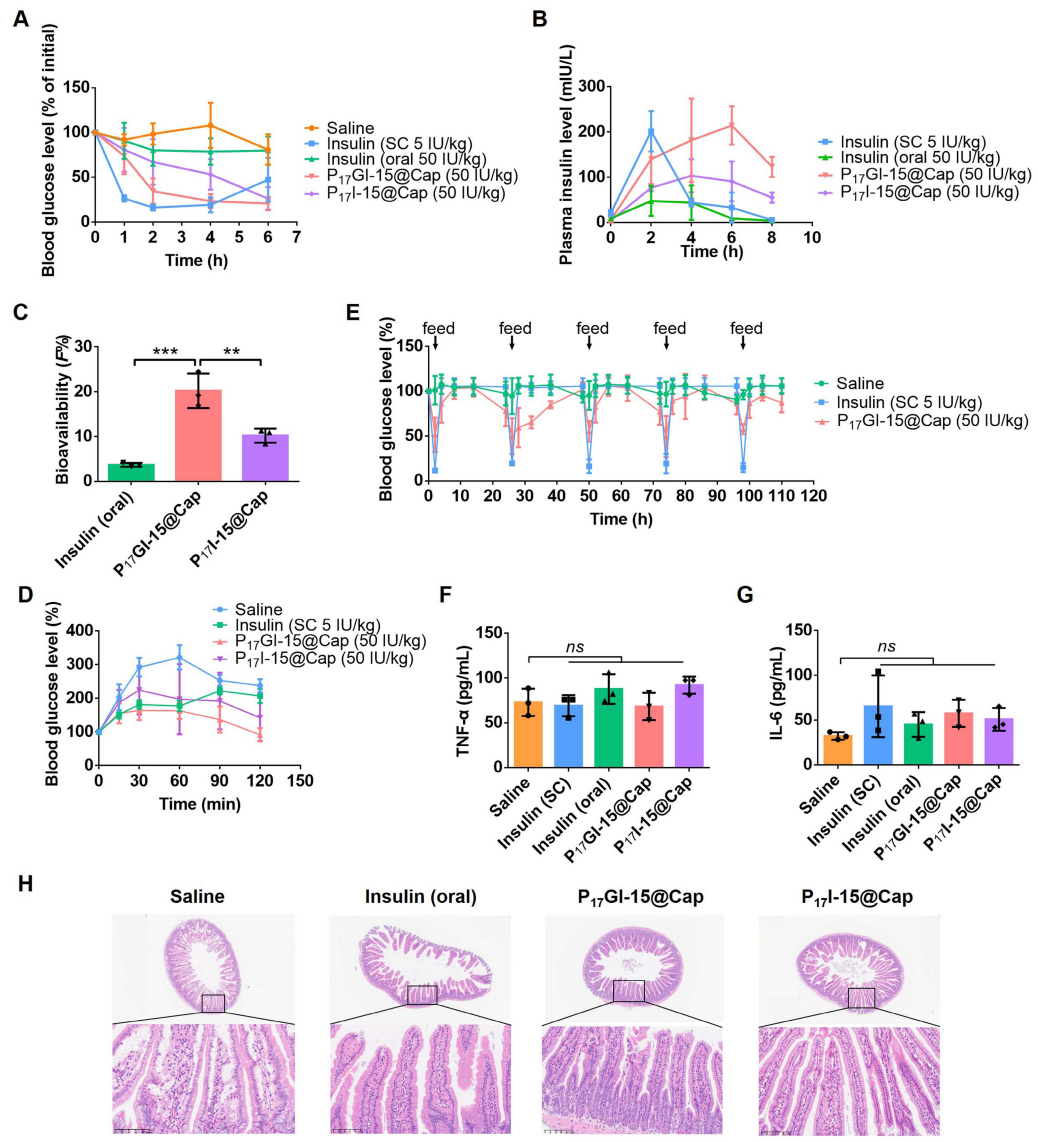
** Pharmacokinetic and hypoglycemic effect of the dual-sensitive NPs* in vivo*. (A)** Variation of blood glucose levels of diabetic mice after treatment for 6 h. Data are presented as the mean ± SD (n = 5). **(B)** Variation of plasma insulin level of diabetic mice after treatment for 8 h (n = 3). **(C)** Oral bioavailability of insulin for different formulations (n = 3).** (D)** IPGTT toward diabetic mice 4 h post-administration (n = 5). **(E)** Variation of blood glucose levels of diabetic mice after administration once a day for five consecutive days (n = 5). After 2 h of administration, mice were re-feeding. **(F)** TNF-α and **(G)** IL-6 in serum collected 24 h after single administration (n = 3). **(H)** Hematoxylin-eosin staining of the small intestines collected 24 h after single administration. Scale bar: 100 μm. Data are presented as the mean ± SD. ns > 0.05, **P < 0.01, ***P < 0.001.

Next, the pharmacokinetic and hypoglycemic effect were evaluated following oral administration of free insulin, P_17_GI-15@Cap and P_17_I-15@Cap in streptozotocin-induced type 1 diabetic mice. As shown in Figure 5A, the subcutaneous administration of insulin at a dose of 5 IU/kg showed a rapid hypoglycemic effect within 1 h, and the average blood glucose value could achieve as low as 16.34% of the initial value at 2 h post injection in diabetic mice. The blood glucose levels increased rapidly after 4 h of administration. Oral administration of free insulin could hardly reduce the blood glucose level at dose of 50 IU/kg, mainly due to the poor oral bioavailability (Figure 5B and 5C). Oral administration of P_17_GI-15@Cap at a dose of 50 IU/kg showed sustained hypoglycemic effect (Figure 5A) and its hypoglycemic effect was better than P_17_I-15@Cap at the same insulin dose. The lowest blood glucose value in P_17_GI-15@Cap group could reach 20.84% of the initial value at 6 h administration, while it was 26.19% for P_17_I-15@Cap at the same time point. The oral bioavailability was 20.24% for P_17_GI-15@Cap, which was obviously higher than that of P_17_I-15@Cap (10.21%). With the same P(HEMA-PBE)-PCB polymer coating, the higher oral bioavailability of dual-sensitive glucose-responsive P_17_GI-15@Cap was mainly attributed to its rapid release ability of insulin at the hyperglycemic level.

An IPGTT was performed to evaluate the effectiveness of the P_17_GI-15@Cap. The glucose solution was injected 4 h post-treatment. As shown in Figure 5D, the blood glucose level was sustainly increased for mice subcutaneously injected of insulin at a dose of 5 IU/kg. In comparison, the blood glucose level was gradually decrease for P_17_GI-15@Cap and P_17_I-15@Cap groups after 30 min administration of glucose. In addition, the hypoglycemic effect of the P_17_GI-15@Cap was better than that of P_17_I-15@Cap.

Furthermore, to verify the therapeutic efficacy after multiple administration, P_17_GI-15@Cap was orally administered to diabetic mice once a day for five consecutive days. After 2 h of administration, mice were re-feeding. As shown in Figure 5E, the blood glucose level decreased after every oral administration. The blood glucose levels of the P_17_GI-15@Cap groups were even lower than that of the subcutaneously administered insulin groups after re-feeding. These results confirmed that the P_17_GI-15@Cap was suitable for continuous treatment.

To evaluate the biocompatibility of P_17_GI-15@Cap, the levels of proinflammatory cytokines TNF-α and IL-6 in serum were measured after 24 h of single and five consecutive days administration, respectively. P_17_GI-15@Cap did not induce any observable increase in terms of TNF-α and IL-6 (Figure 5F, 5G and Figure S22). Furthermore, hematoxylin-eosin staining was performed on the small intestine after 24 h of single and five consecutive days administration, respectively. As shown in Figure 5H and Figure S23, there was no inflammation and pathological changes of the small intestines during oral treatment, indicating that P_17_GI-15@Cap had good biocompatibility for *in vivo* application.

## Conclusion

Oral administration is considered to be the most convenient and safe choice for patients in clinical. However, the oral bioavailability of insulin is very low due to the poor oral absorption into blood circulation. To overcome the physiological barriers in GI tract and achieve enhanced and safe glucose-responsive oral insulin delivery, we developed pH and H_2_O_2_ dual-sensitive PGI NPs. With the modification of zwitterionic PCB, the PGI NPs could enhance the transport of insulin across the intestinal epithelium due to its binding ability with PAT1 on epithelial cell layer. By virtue of the generated low pH and high H_2_O_2_ with GOx in hyperglycemic environment, the dual-sensitive PGI NPs were disassembled to achieve rapid and sustained release of insulin. After oral administration of the dual-sensitive NPs in enteric capsules into T1DM mouse model, the oral bioavailability of insulin reached 20.24%, and the NPs achieved hypoglycemic effect for a few hours longer than subcutaneously injected insulin. Importantly, the pH and H_2_O_2_ dual-sensitive NPs could ameliorate the local decline of pH and rise of H_2_O_2_ to avoid the toxic side effect. Therefore, this work would provide a promising platform for the enhanced and safe treatment of diabetes mellitus.

## Supplementary Material

Supplementary figures.

## References

[1] Li Y, Zhang Y, Niu T, Pang Y, Shi Y, Zeng Q (2023). Discovery and development of tricyclic matrinic derivatives as anti-diabetic candidates by AMPKα activation. Chin Chem Lett.

[2] GhavamiNejad A, Li J, Lu B, Zhou L, Lam L, Giacca A (2019). Glucose-responsive composite microneedle patch for hypoglycemia-triggered delivery of native glucagon. Adv Mater.

[3] Xiao Y, Sun H, Du J (2017). Sugar-breathing glycopolymersomes for regulating glucose level. J Am Chem Soc.

[4] Chen Y, Li P, Modica JA, Drout RJ, Farha OK (2018). Acid-resistant mesoporous metal-organic framework toward oral insulin delivery: protein encapsulation, protection, and release. J Am Chem Soc.

[5] Brown TD, Whitehead KA, Mitragotri S (2019). Materials for oral delivery of proteins and peptides. Nat Rev Mater.

[6] Qin X, Yu C, Wei J, Li L, Zhang C, Wu Q (2019). Rational design of nanocarriers for intracellular protein delivery. Adv Mater.

[7] Xu R, Bhangu SK, Sourris KC, Vanni D, Sani MA, Karas JA (2023). An engineered nanosugar enables rapid and sustained glucose-responsive insulin delivery in diabetic mice. Adv Mater.

[8] Wang J, Yu J, Zhang Y, Zhang X, Kahkoska AR, Chen G (2019). Charge-switchable polymeric complex for glucose-responsive insulin delivery in mice and pigs. Sci Adv.

[9] Yu J, Zhang Y, Wang J, Wen D, Kahkoska AR, Buse JB (2018). Glucose-responsive oral insulin delivery for postprandial glycemic regulation. Nano Res.

[10] Xiao Y, Tang Z, Wang J, Liu C, Kong N, Farokhzad OC (2020). Oral insulin delivery platforms: strategies to address the biological barriers. Angew Chem Int Ed Engl.

[11] Sun M, Hu H, Sun L, Fan Z (2020). The application of biomacromolecules to improve oral absorption by enhanced intestinal permeability: a mini-review. Chin Chem Lett.

[12] Zelikin AN, Ehrhardt C, Healy AM (2016). Materials and methods for delivery of biological drugs. Nat Chem.

[13] Ito S, Torii Y, Chikamatsu S, Harada T, Yamaguchi S, Ogata S (2021). Oral coadministration of Zn-insulin with d-form small intestine-permeable cyclic peptide enhances its blood glucose-lowering effect in mice. Mol Pharm.

[14] Li Y, Zhang W, Zhao R, Zhang X (2022). Advances in oral peptide drug nanoparticles for diabetes mellitus treatment. Bioact Mater.

[15] Li Y, Ji W, Peng H, Zhao R, Zhang T, Lu Z (2021). Charge-switchable zwitterionic polycarboxybetaine particle as an intestinal permeation enhancer for efficient oral insulin delivery. Theranostics.

[16] Zhou Y, Chen Z, Zhao D, Li D, He C, Chen X (2021). A pH-triggered self-unpacking capsule containing zwitterionic hydrogel-coated mof nanoparticles for efficient oral exendin-4 delivery. Adv Mater.

[17] Fang H, Chen L, Deng Z, Gao Y, Yang Y, Chen Q (2023). In situ polymerization of zwitterions on therapeutic proteins to enable their effective oral delivery. ACS Nano.

[18] Sonaje K, Lin KJ, Wang JJ, Mi FL, Chen CT, Juang JH (2010). Self-assembled pH-sensitive nanoparticles: a platform for oral delivery of protein drugs. Adv Funct Mater.

[19] Zhu X, Wu J, Shan W, Zhou Z, Liu M, Huang Y (2016). Sub-50 nm nanoparticles with biomimetic surfaces to sequentially overcome the mucosal diffusion barrier and the epithelial absorption barrier. Adv Funct Mater.

[20] Fan W, Xia D, Zhu Q, Li X, He S, Zhu C (2018). Functional nanoparticles exploit the bile acid pathway to overcome multiple barriers of the intestinal epithelium for oral insulin delivery. Biomaterials.

[21] Volpatti LR, Facklam AL, Cortinas AB, Lu Y-C, Matranga MA, MacIsaac C (2021). Microgel encapsulated nanoparticles for glucose-responsive insulin delivery. Biomaterials.

[22] Lim ZW, Ping Y, Miserez A (2018). Glucose-responsive peptide coacervates with high encapsulation efficiency for controlled release of insulin. Bioconjug Chem.

[23] Yu J, Qian C, Zhang Y, Cui Z, Zhu Y, Shen Q (2017). Hypoxia and H_2_O_2_ dual-sensitive vesicles for enhanced glucose-responsive insulin delivery. Nano Lett.

[24] Volpatti LR, Matranga MA, Cortinas AB, Delcassian D, Daniel KB, Langer R (2019). Glucose-responsive nanoparticles for rapid and extended self-regulated insulin delivery. ACS Nano.

[25] Hu X, Yu J, Qian C, Lu Y, Kahkoska AR, Xie Z (2017). H_2_O_2_-responsive vesicles integrated with transcutaneous patches for glucose-mediated insulin delivery. ACS Nano.

[26] Shan W, Zhu X, Tao W, Cui Y, Liu M, Wu L (2016). Enhanced oral delivery of protein drugs using zwitterion-functionalized nanoparticles to overcome both the diffusion and absorption barriers. ACS Appl Mater Interfaces.

[27] Han X, Lu Y, Xie J, Zhang E, Zhu H, Du H (2020). Zwitterionic micelles efficiently deliver oral insulin without opening tight junctions. Nat Nanotechnol.

[28] Li Y, Cheng Q, Jiang Q, Huang Y, Liu H, Zhao Y (2014). Enhanced endosomal/lysosomal escape by distearoyl phosphoethanolamine-polycarboxybetaine lipid for systemic delivery of siRNA. J Control Release.

[29] Li Y, Li Y, Ji W, Lu Z, Liu L, Shi Y (2018). Positively charged polyprodrug amphiphiles with enhanced drug loading and reactive oxygen species-responsive release ability for traceable synergistic therapy. J Am Chem Soc.

[30] Li M, Zhang W, Li J, Qi Y, Peng C, Wang N (2023). Zwitterionic polymers: addressing the barriers for drug delivery. Chin Chem Lett.

[31] Sonaje K, Chen Y-J, Chen H-L, Wey S-P, Juang J-H, Nguyen H-N (2010). Enteric-coated capsules filled with freeze-dried chitosan/poly(γ-glutamic acid) nanoparticles for oral insulin delivery. Biomaterials.

[32] Lai SK, Wang Y-Y, Hanes J (2009). Mucus-penetrating nanoparticles for drug and gene delivery to mucosal tissues. Adv Drug Deliv Rev.

[33] Shan W, Zhu X, Liu M, Li L, Zhong J, Sun W (2015). Overcoming the diffusion barrier of mucus and absorption barrier of epithelium by self-assembled nanoparticles for oral deliveryof insulin. ACS Nano.

[34] Pereira de Sousa I, Steiner C, Schmutzler M, Wilcox MD, Veldhuis GJ, Pearson JP (2015). Mucus permeating carriers: formulation and characterization of highly densely charged nanoparticles. Eur J Pharm Biopharm.

[35] Gu Z, Dang TT, Ma M, Tang BC, Cheng H, Jiang S (2013). Glucose-responsive microgels integrated with enzyme nanocapsules for closed-loop insulin delivery. ACS Nano.

[36] Xu Y, Xu J, Shan W, Liu M, Cui Y, Li L (2016). The transport mechanism of integrin αvβ3 receptor targeting nanoparticles in Caco-2 cells. Int J Pharm.

[37] Yun Y, Cho YW, Park K (2013). Nanoparticles for oral delivery: targeted nanoparticles with peptidic ligands for oral protein delivery. Adv Drug Deliv Rev.

[38] Yin L, Song Z, Kim KH, Zheng N, Tang H, Lu H (2013). Reconfiguring the architectures of cationic helical polypeptides to control non-viral gene delivery. Biomaterials.

