# Supplementary material for: pH and H2O2 dual-sensitive nanoparticles enable enhanced and safe glucose-responsive oral insulin delivery for diabetes mellitus treatment
Source: Theranostics. 2024 Sep 3;14(14):5596–607. doi: 10.7150/thno.98177 (PMC11413777; doi:10.7150/thno.98177)
Supplement: Supplementary file 1 — Supplementary figures. [file thnov14p5596s1.pdf]

Supplementary Material

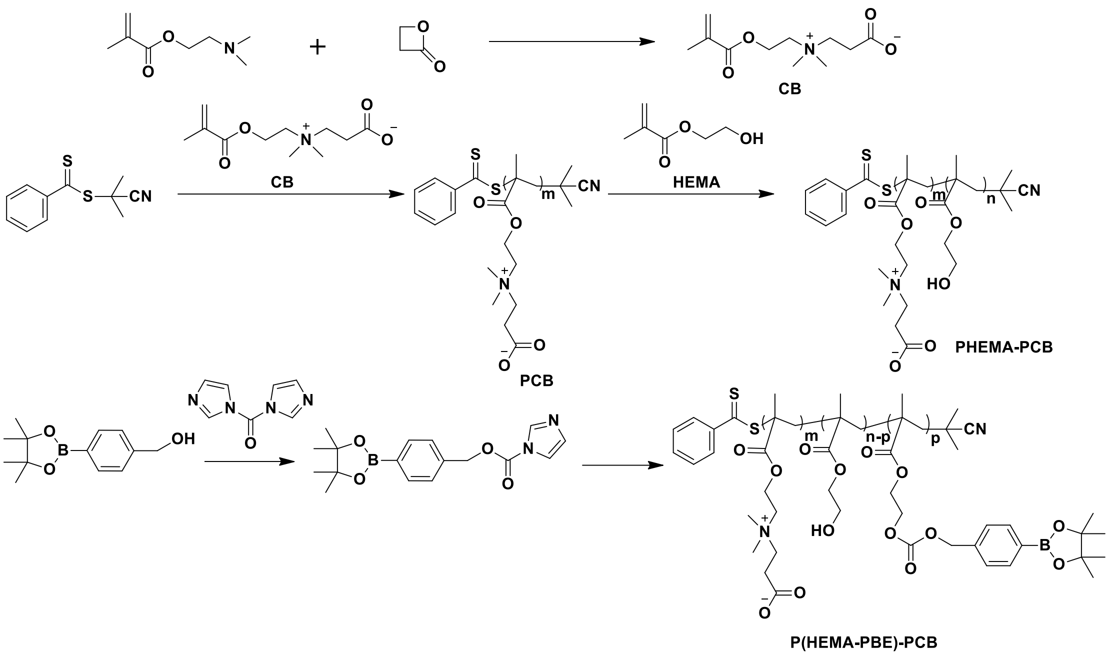

Figure S1. The synthetic route of P(HEMA-PBE)-PCB.

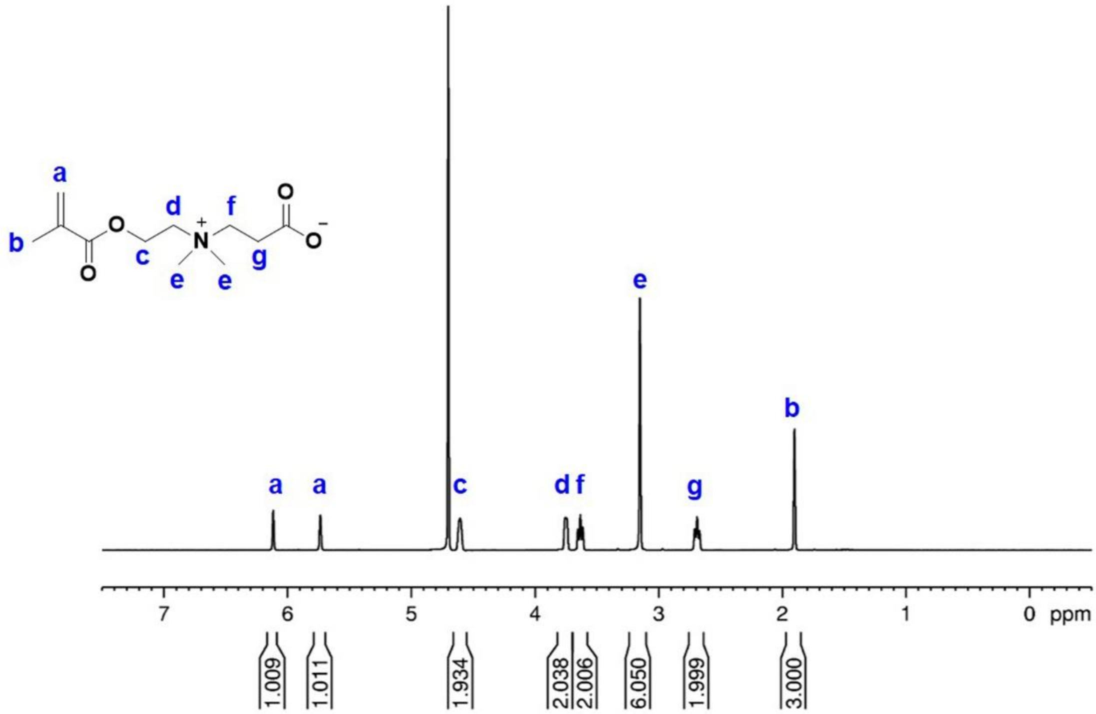

Figure S2. ^{1}H NMR spectrum of CB monomer (D_2O).

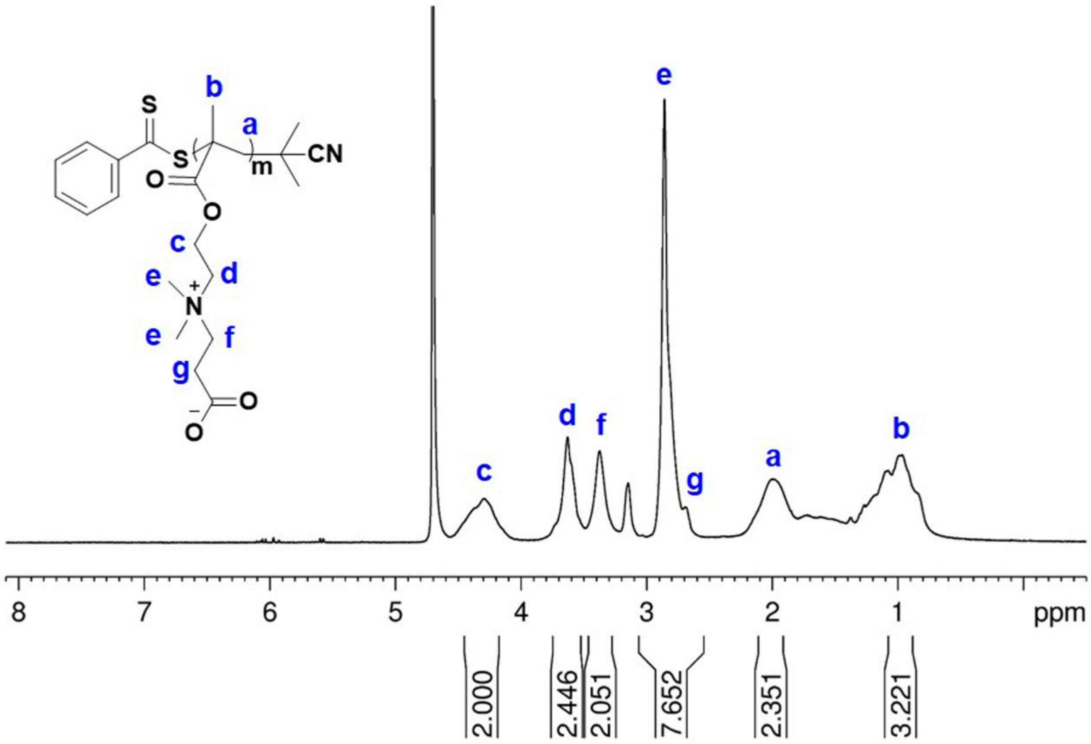

Figure S3. ^{1}H NMR spectrum of PCB polymer (D_2O).

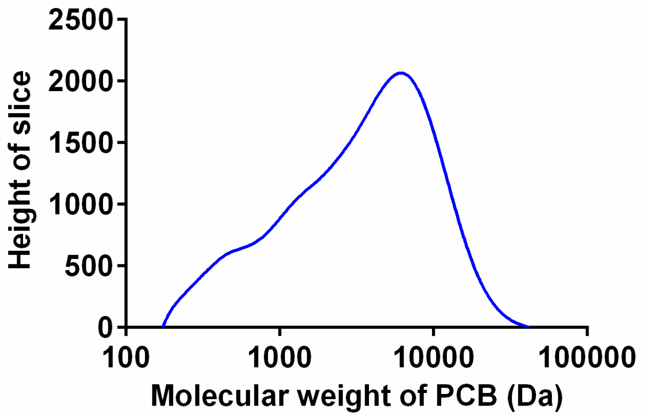

Figure S4. The molecular weight of PCB measured via GPC.

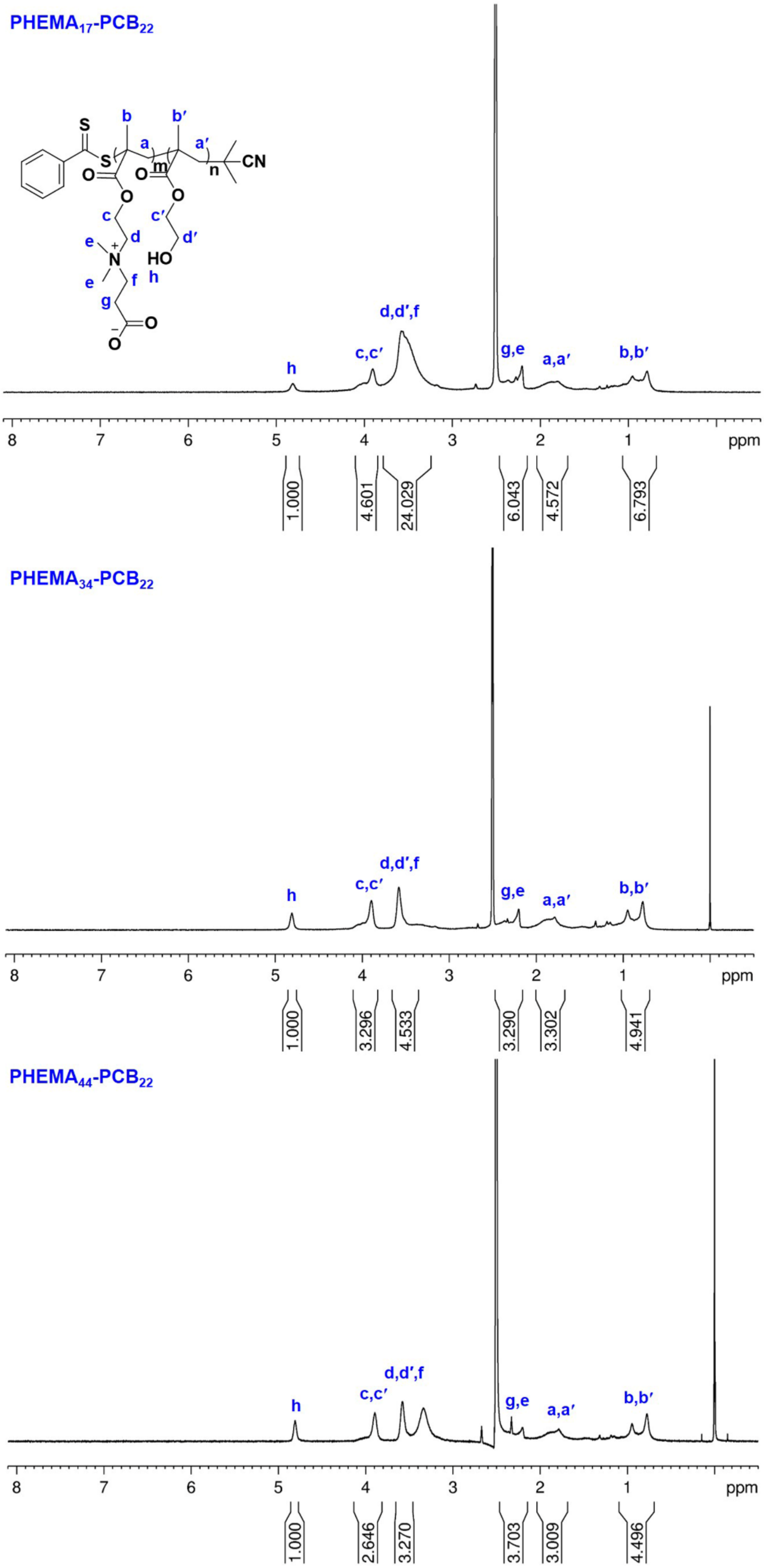

Figure S5. ^{1}H NMR spectra of PHEMA-PCB polymers (DMSO-d$_6$).

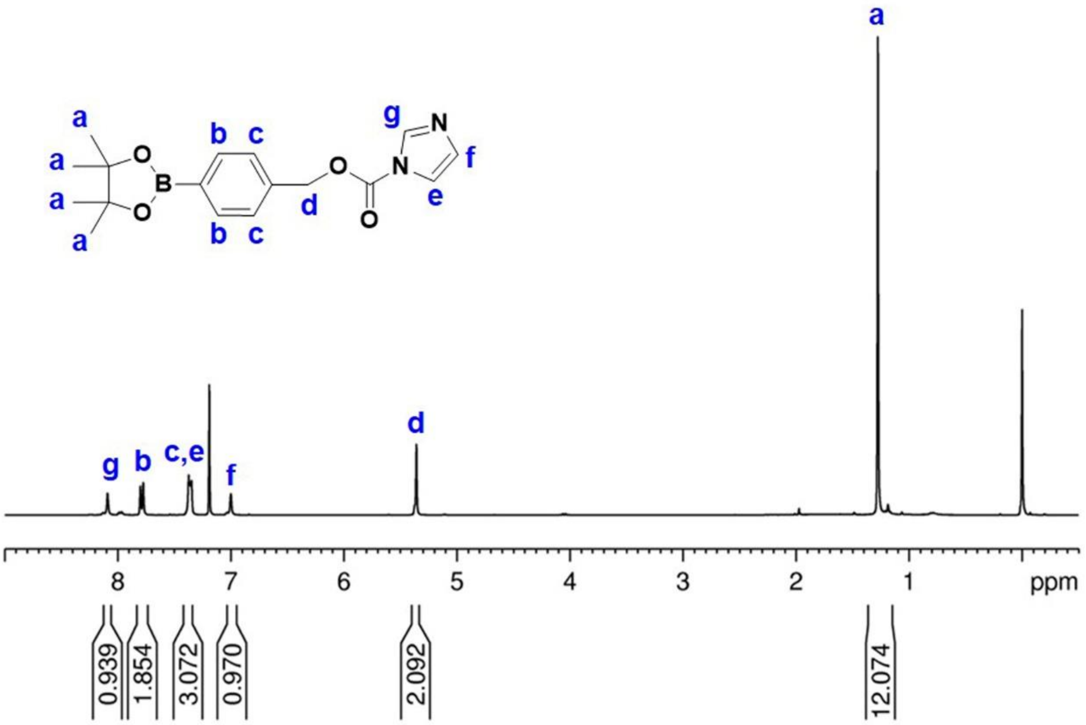

Figure S6. ^{1}H NMR spectrum of 4-(imidazoyl carbamate)phenylboronic acid pinacol ester ($CDCl_3$).

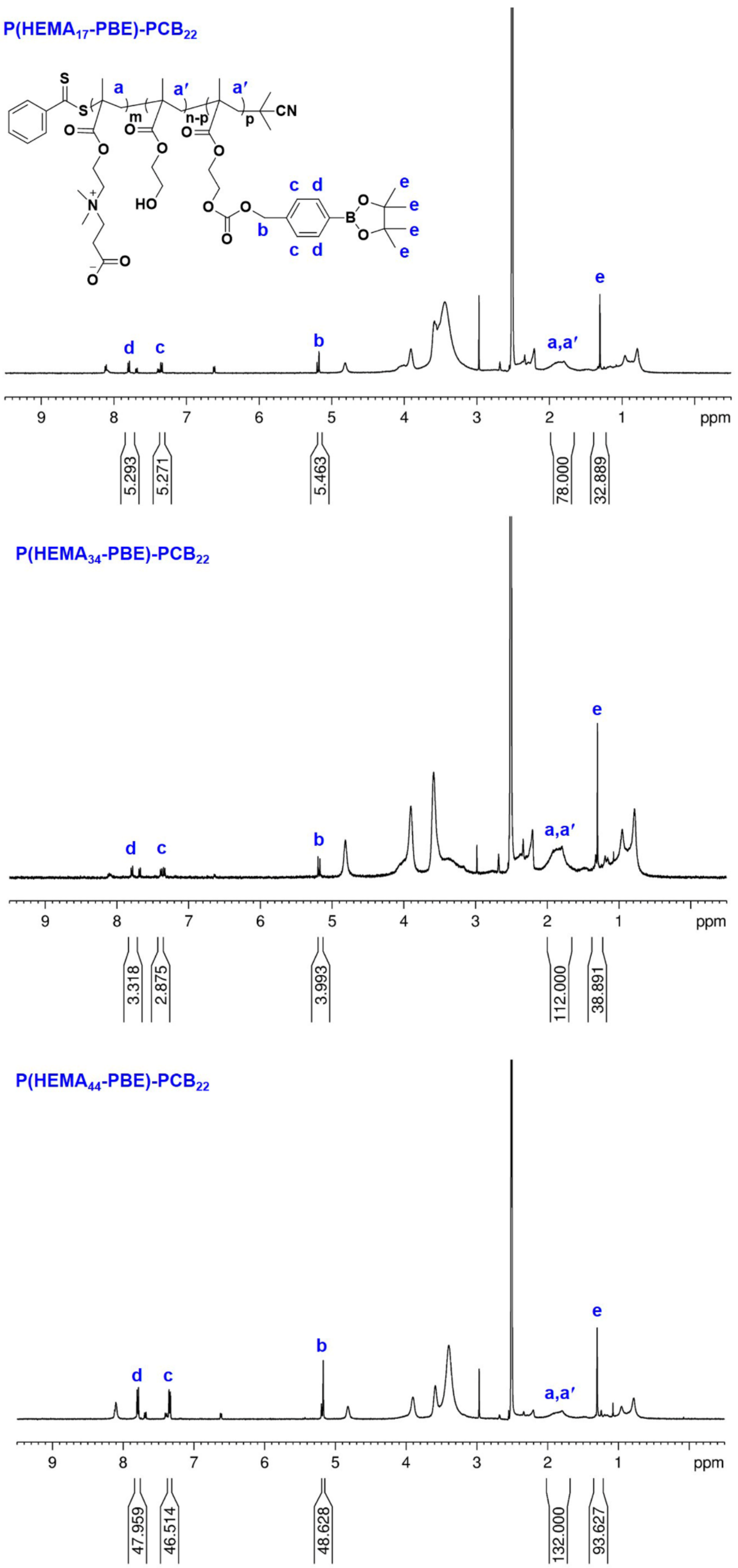

Figure S7. ^{1}H NMR spectra of P(HEMA-PBE)-PCB polymers (DMSO-d$_6$).

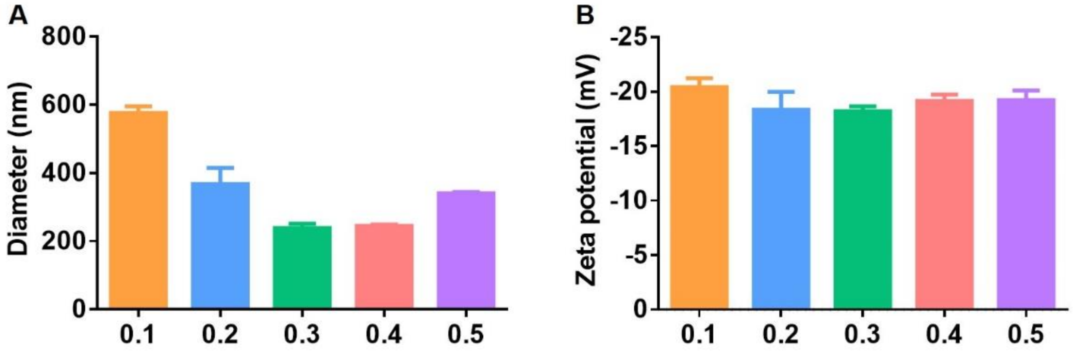

Figure S8. (A) Hydrodynamic diameter and (B) zeta potential of precipitates. $ZnCl_2$ solution was slowly added to the mixture of insulin and GOx under stirring at various mass ratios between $ZnCl_2$ and insulin. Data are presented as the mean ± SD (n = 3).

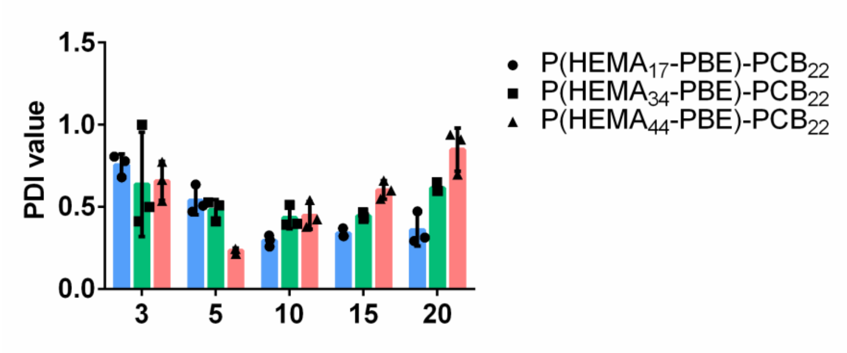

Figure S9. PDI value of PGI NPs with different mass ratios between P(HEMA-PBE)-PCB and insulin.

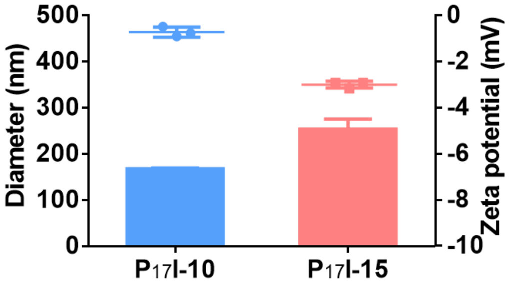

Figure S10. The hydrodynamic diameter and zeta potential of P_{17}I-10 and P_{17}I-15 NPs. Data are presented as the mean ± SD (n = 3).

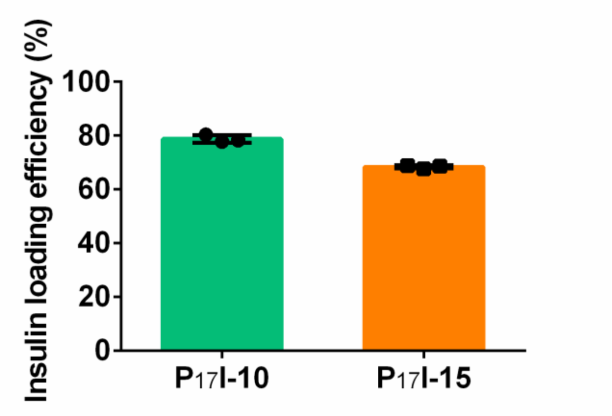

Figure S11. Insulin loading efficiency of P_{17}I-10 and P_{17}I-15 NPs. Data are presented as the mean ± SD (n = 3).

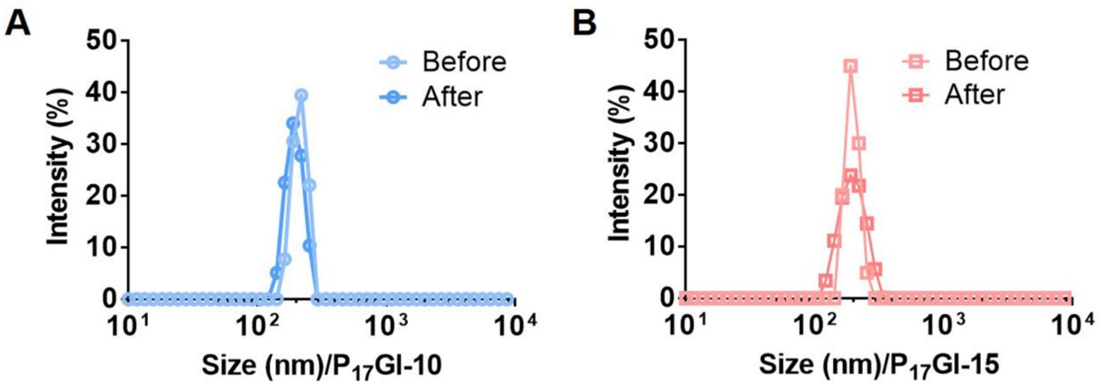

Figure S12. Size distribution of (A) P_{17}GI-10 and (B) P_{17}GI-15 NPs before and after lyophilization.

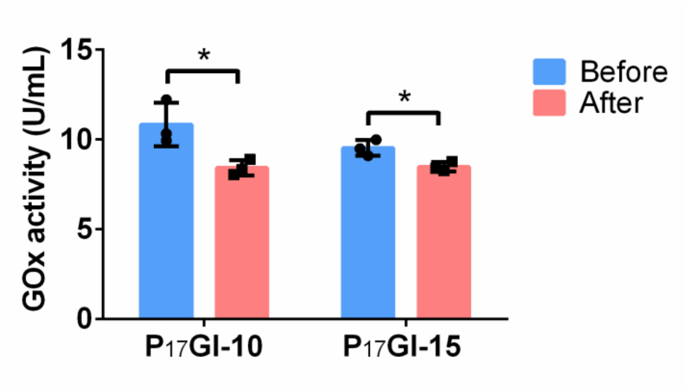

Figure S13. GOx activity of NPs before and after lyophilization. The concentration of GOx was 62.5 μg/mL. Data are presented as the mean ± SD (n = 3). **P* < 0.05.

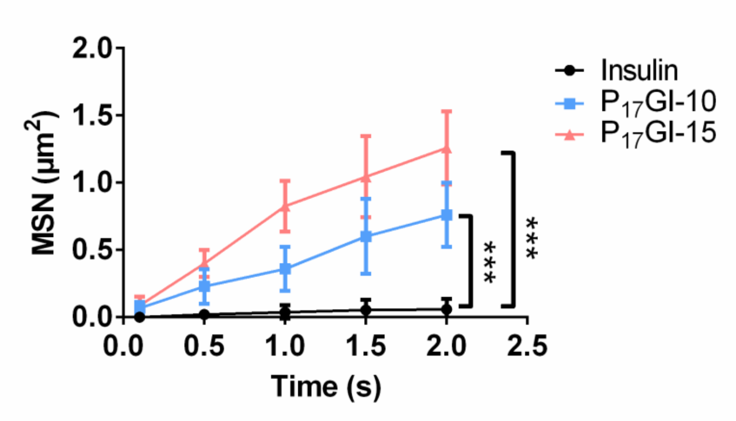

Figure S14. MSD calculated from Figure 1H. Data are presented as the mean ± SD (n = 3). ****P* < 0.001.

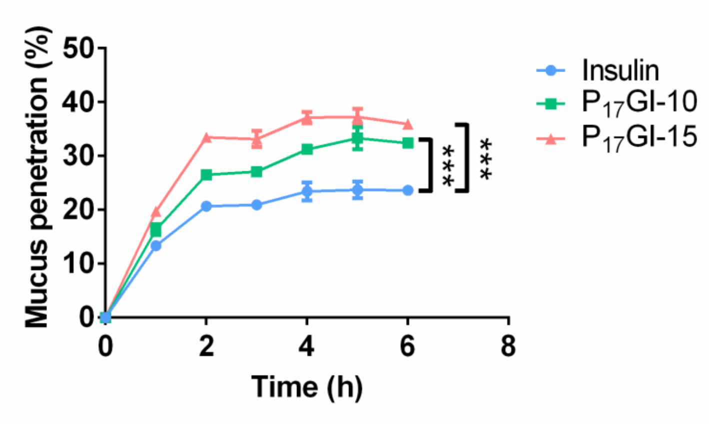

Figure S15. The percentage of Cy5-insulin across the mucus in transwell. Data are presented as the mean ± SD (n = 3). ***P < 0.001.

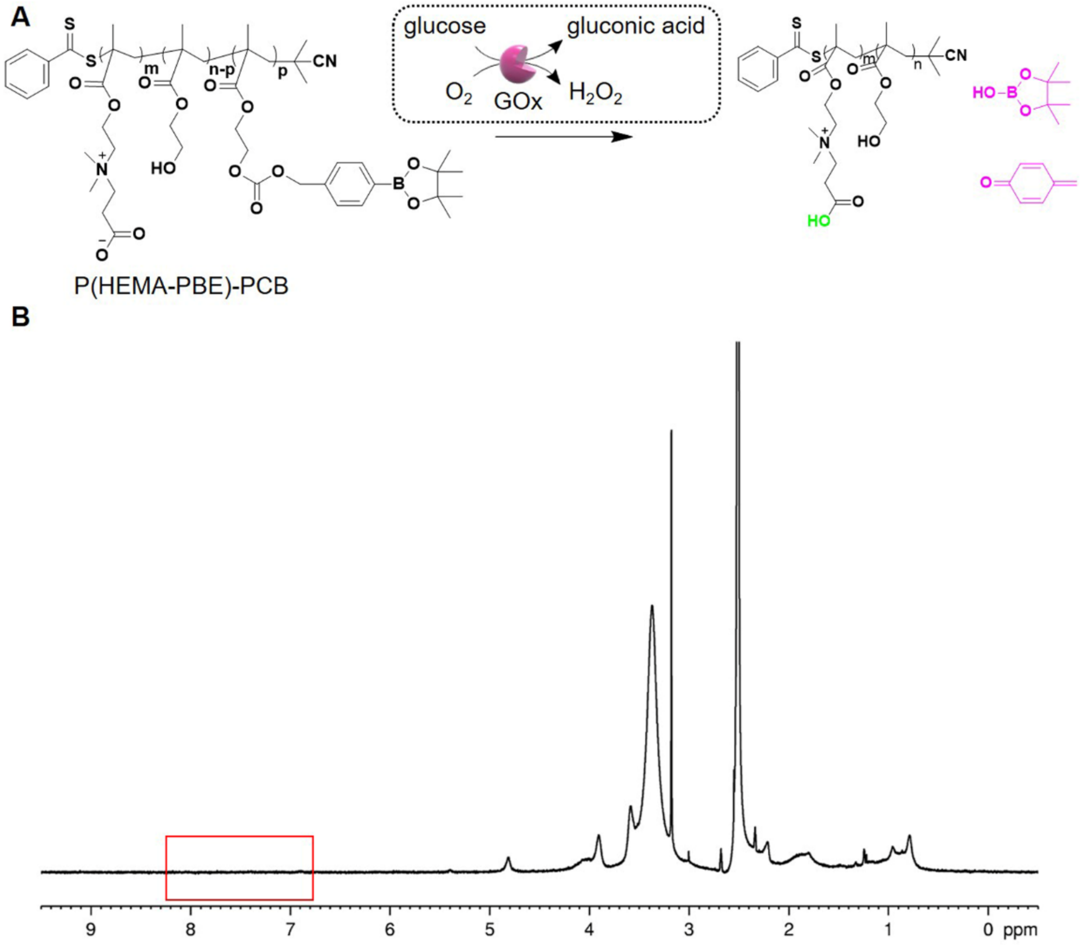

Figure S16. (A) Mechanism of the glucose-responsive P(HEMA-PBE)-PCB polymer. (B) ^{1}H NMR spectrum of P(HEMA$_{17}$-PBE)-PCB$_{22}$ polymer after incubating in H_2O_2 solution for 24 h.

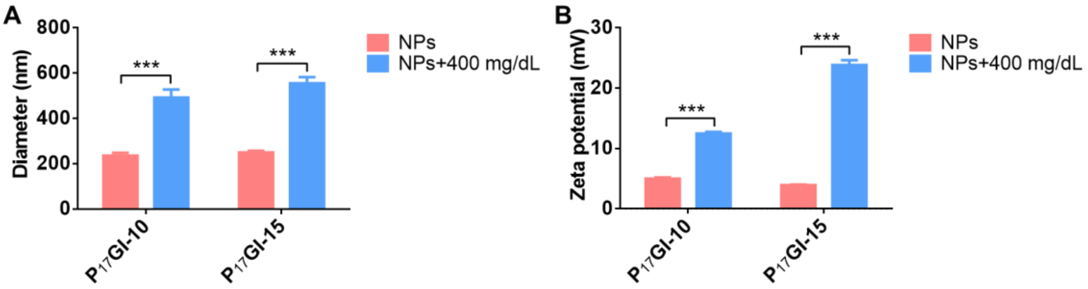

Figure S17. The changes of (A) diameter and (B) zeta potential of P_{17}GI-10 and P_{17}GI-15 NPs after incubating in 400 mg/dL glucose solution for 2 h. Data are presented as the mean ± SD (n = 3). ****P* < 0.001.

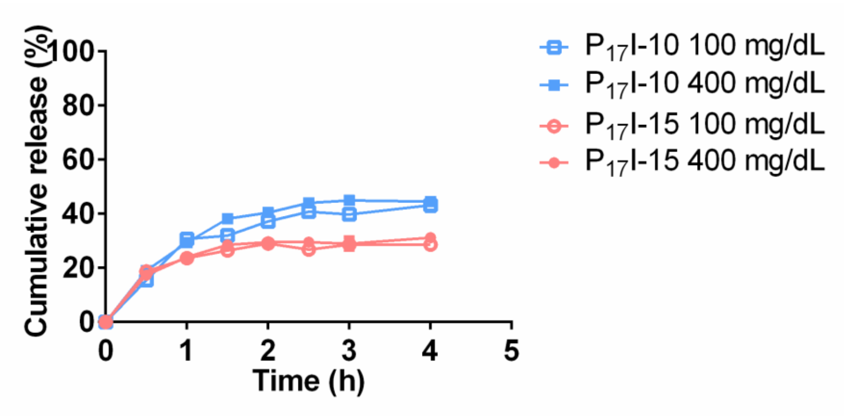

Figure S18. Cumulative insulin release of P_{17}I-10 and P_{17}I-15 NPs in 100 mg/dL and 400 mg/dL glucose solution, respectively. Data are presented as the mean ± SD (n = 3).

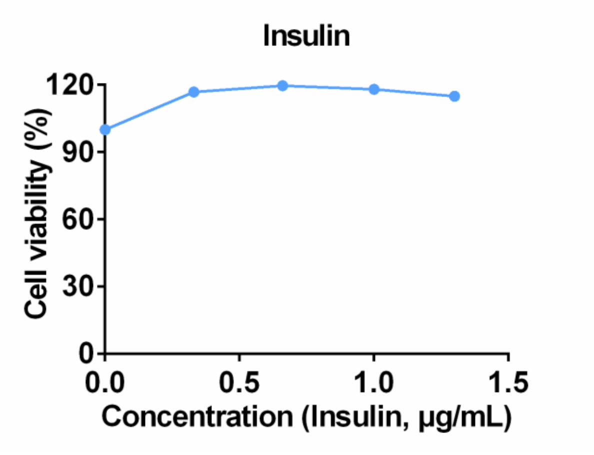

Figure S19. The cell viability of Caco-2 cells after incubating with insulin for 24 h. Data are presented as the mean ± SD (n = 3).

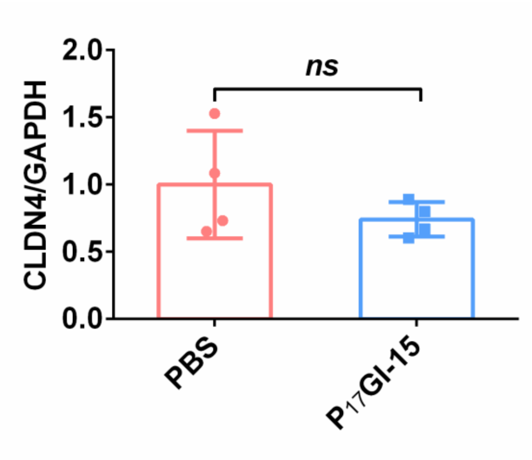

Figure S20. Quantified results of CLDN4 in Figure 3D using ImageJ. Data are presented as the mean ± SD (n = 4). ns > 0.05.

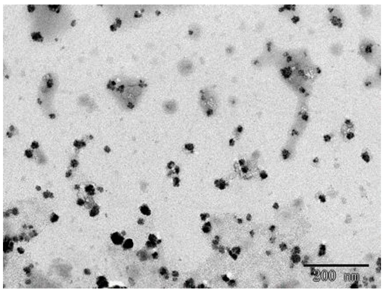

Figure S21. TEM image of P_{17}GI-15 NPs across the Caco-2 cell lay in transwell. Scale bar: 200 nm.

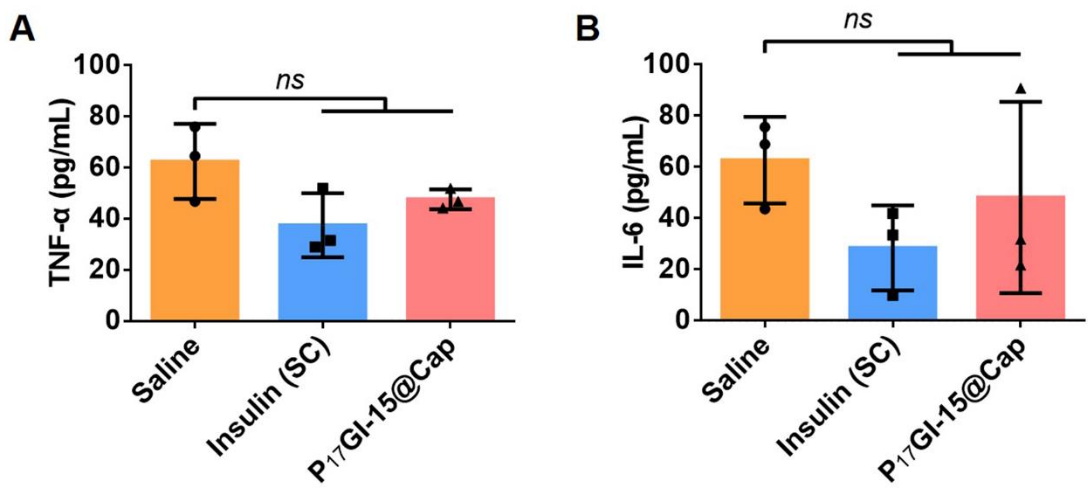

Figure S22. (A) TNF-α and (B) IL-6 in serum collected 24 h after five consecutive days administration. Data are presented as the mean ± SD (n = 3). ns > 0.05.

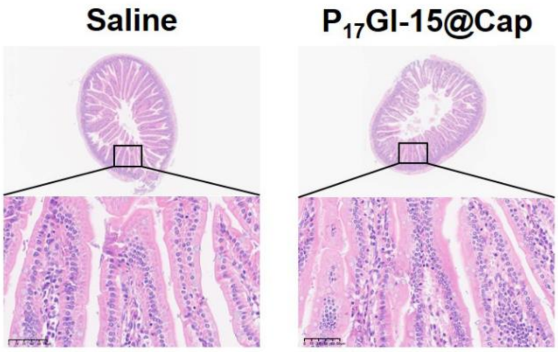

Figure S23. Hematoxylin-eosin staining of the small intestines collected 24 h after five consecutive days administration. Scale bar: 50 μm.
